# Current and Emerging Diagnostic Approaches for Tuberculosis in People Living with HIV: Challenges and Future Perspectives

**DOI:** 10.3390/tropicalmed11080213

**Published:** 2026-07-28

**Authors:** Guillermo Eduardo Cuéllar-Nevárez, José Rafael Linares Morales, Luis Arturo Camacho Silvas, Sandra Guadalupe Chavarría Hidalgo, Guadalupe Virginia Nevárez-Moorillón, Karla Elva Loza Solano

**Affiliations:** 1 Licenciatura en Salud Pública, Facultad de Medicina y Ciencias Biomédicas, Universidad Autónoma de Chihuahua, Circuito Universitario S/N, Chihuahua 31109, Mexico; p319461@uach.mx (J.R.L.M.); lsilvas@uach.mx (L.A.C.S.); schavarria@uach.mx (S.G.C.H.); kloza@uach.mx (K.E.L.S.); 2 Facultad de Ciencias Químicas, Universidad Autónoma de Chihuahua, Circuito Universitario S/N, Chihuahua 31125, Mexico; vnevare@uach.mx

**Keywords:** tuberculosis, HIV, HIV-associated tuberculosis, tuberculosis diagnosis, Xpert MTB/RIF Ultra, lipoarabinomannan, FujiLAM, host biomarkers, artificial intelligence, diagnostic algorithms

## Abstract

Tuberculosis (TB) remains a leading cause of morbidity and mortality among people living with human immunodeficiency virus (HIV), in whom immunosuppression often results in atypical clinical manifestations, paucibacillary disease, extrapulmonary involvement, and disseminated forms that complicate timely diagnosis. This narrative review summarizes current and emerging diagnostic approaches for TB-HIV, with emphasis on their diagnostic performance, clinical applicability, implementation challenges, and potential integration into future algorithms. Conventional methods, including sputum smear microscopy, mycobacterial culture, and chest radiography, remain relevant but have important limitations in people living with HIV (PLHIV), particularly in advanced immunosuppression. Rapid molecular tests, especially Xpert MTB/RIF and Xpert MTB/RIF Ultra, have improved early detection of *Mycobacterium tuberculosis* and rifampicin resistance, although diagnostic gaps persist in sputum-scarce and extrapulmonary disease. Non-sputum-based tests, particularly WHO-recommended urine LF-LAM and emerging next-generation assays such as FujiLAM II, offer important opportunities for severely ill patients and those with advanced HIV disease. Host-derived biomarkers, transcriptomic signatures, and artificial intelligence-assisted chest imaging may further strengthen screening, triage, and diagnostic prioritization. Integrated, context-adapted diagnostic algorithms combining microbiological, molecular, urinary, imaging, and host-response tools are needed to improve early TB detection and reduce mortality in resource-limited settings.

## 1. Introduction

Tuberculosis (TB) remains one of the most important infectious diseases worldwide and represents a significant cause of morbidity and mortality, particularly in low- and middle-income countries. According to the World Health Organization Global Tuberculosis Report 2025, an estimated 10.7 million people developed TB in 2024, of whom 8.3 million were officially diagnosed and notified. Although incident cases decreased slightly compared with 2023, the global burden of disease remains high, and important gaps persist in timely access to diagnosis. In addition, TB caused approximately 1.23 million deaths in 2024, including 150,000 deaths among people living with human immunodeficiency virus (HIV) [1].

Although global TB notification has progressively recovered after the coronavirus disease 2019 (COVID-19) pandemic, the diagnostic gap remains considerable. In 2024, the difference between estimated incident cases and officially diagnosed and notified cases was approximately 2.4 million people. This gap is especially relevant in populations at increased risk of severe disease or delayed diagnosis, such as people living with HIV (PLHIV), in whom TB may present as paucibacillary, extrapulmonary, or disseminated disease [1,2].

HIV infection is one of the main risk factors for the development of active TB. Progressive HIV-induced immunosuppression favors both the reactivation of latent *Mycobacterium tuberculosis* infection and rapid progression to active disease after recent infection [3]. Consequently, PLHIV have a substantially higher risk of developing TB than the general population. Although universal access to antiretroviral therapy (ART) has markedly reduced HIV-related morbidity and mortality, advanced HIV disease remains common in many settings because of late HIV diagnosis, delayed ART initiation, treatment interruption, virological failure, and persistent inequities in access to care. Consequently, TB remains the leading cause of HIV-associated death worldwide and continues to represent one of the principal opportunistic infections requiring early diagnosis and prompt treatment [2,4].

TB-HIV coinfection poses major clinical and public health challenges. In addition to increasing mortality and therapeutic complexity, it substantially modifies the clinical presentation of disease. Patients with advanced immunosuppression often present with paucibacillary disease, extrapulmonary involvement, or disseminated TB, which reduces the diagnostic performance of conventional methods based on direct microbiological detection [3]. These characteristics contribute to diagnostic delays, late treatment initiation, and poorer clinical outcomes.

For more than a century, sputum smear microscopy, mycobacterial culture, and chest radiography constituted the fundamental pillars of TB diagnosis [5,6]. However, the limited sensitivity of smear microscopy, particularly in patients with HIV and paucibacillary disease, as well as the prolonged time required for culture, have driven the incorporation of rapid diagnostic tests and methods that do not rely exclusively on sputum [5,7,8].

Among the most relevant innovations are the Xpert MTB/RIF and Xpert MTB/RIF Ultra platforms, currently recommended by the World Health Organization as initial diagnostic tests in multiple clinical contexts. These technologies allow the detection of *M. tuberculosis* DNA and rifampicin resistance in a much shorter time than culture, with greater sensitivity than smear microscopy, especially in settings of paucibacillary disease or HIV coinfection [5,8]. Likewise, the development of urine-based lipoarabinomannan tests has opened new opportunities for TB diagnosis in patients with advanced immunosuppression. LF-LAM is currently recommended for defined clinical scenarios in PLHIV, whereas next-generation assays such as FujiLAM II remain under evaluation as potentially more sensitive and reproducible alternatives [5,9,10].

In parallel, recent advances in host biomarkers, transcriptomic signatures, and artificial intelligence applied to medical imaging are transforming the TB diagnostic landscape [11,12,13,14]. These emerging technologies have the potential to complement current algorithms as tools for screening, triage, or diagnostic prioritization, especially in resource-limited settings where barriers to timely access to molecular tests, culture, or specialized evaluation persist [2,11,12,13].

Therefore, an updated synthesis of available and developing diagnostic tools for TB detection in PLHIV is needed. The objective of this narrative review is to analyze conventional diagnostic methods, currently recommended molecular technologies, and emerging approaches for TB diagnosis in this population, highlighting their strengths, limitations, and future perspectives for improving early disease detection.

## 2. Approach to the Narrative Review

This review was developed as a narrative, state-of-the-art review aimed at synthesizing current and emerging advances in the diagnosis of TB in people living with human immunodeficiency virus (HIV). The objective was not to conduct a systematic review or meta-analysis, but rather to critically integrate relevant evidence from international guidelines, systematic reviews, meta-analyses, clinical trials, diagnostic accuracy studies, and recent articles on conventional, molecular, urinary, immunological, radiological, and digital diagnostic technologies.

The literature was selected with emphasis on publications related to TB-HIV, rapid molecular diagnosis, Xpert MTB/RIF, Xpert Ultra, urine-based lipoarabinomannan tests, FujiLAM, host biomarkers, artificial intelligence-assisted chest radiography, extrapulmonary TB, and integrated diagnostic algorithms. Priority was given to World Health Organization documents, Cochrane reviews, individual participant data meta-analyses, pragmatic clinical trials, and studies published in peer-reviewed journals, especially those providing information specific to PLHIV or populations with paucibacillary, extrapulmonary, or disseminated disease.

The evidence was organized according to the type of diagnostic technology and its clinical applicability in different scenarios: outpatients, hospitalized patients, people with advanced HIV disease, suspected extrapulmonary TB, disseminated disease, and risk of drug resistance. Owing to the narrative nature of this review, no formal risk-of-bias assessment or original quantitative synthesis was performed; however, an effort was made to distinguish between technologies recommended by international guidelines, tools with consolidated clinical evidence, and emerging approaches that still require prospective validation or programmatic evaluation.

To support the narrative synthesis, relevant literature was identified through searches of PubMed/MEDLINE, Cochrane Library, and official World Health Organization documents, complemented by reference checking of key reviews, guidelines, meta-analyses, and diagnostic accuracy studies. Search terms included combinations of “tuberculosis,” “HIV,” “people living with HIV,” “TB diagnosis,” “Xpert MTB/RIF,” “Xpert Ultra,” “lipoarabinomannan,” “LF-LAM,” “FujiLAM,” “host biomarkers,” “C-reactive protein,” “transcriptomic signatures,” “computer-aided detection,” “artificial intelligence,” “extrapulmonary tuberculosis,” and “disseminated tuberculosis.” Priority was given to publications with direct relevance to TB diagnosis in PLHIV, advanced HIV disease, paucibacillary TB, extrapulmonary TB, disseminated disease, or implementation in high-burden and resource-limited settings.

During manuscript preparation, the authors used ChatGPT (OpenAI; GPT-5.5 Thinking and GPT-5.6 Thinking) to support language translation, academic editing, improvement of technical clarity, manuscript structure, and consistency checks. The tool was also used to assist in identifying possible redundancies, inconsistencies in citation placement, and issues requiring author verification. No generative artificial intelligence tool was used to perform literature searches independently, select studies, extract data, conduct analyses, generate original scientific evidence, or make final interpretative decisions. All AI-assisted outputs were critically reviewed, edited, and verified by the authors, who take full responsibility for the content, accuracy, integrity, and final version of the manuscript.

## 3. Diagnostic Challenges of TB in People Living with HIV

The diagnosis of TB in PLHIV remains complex because immunosuppression modifies the clinical, microbiological, and radiological presentation of the disease. Unlike classic pulmonary tuberculosis (PTB) observed in immunocompetent individuals, TB-HIV may present with nonspecific symptoms, lower bacillary load in sputum, atypical radiographs, extrapulmonary forms, and disseminated disease. This heterogeneity reduces the usefulness of diagnostic algorithms focused exclusively on chronic cough, sputum smear microscopy, and respiratory samples, especially in patients with advanced immunosuppression [2,7].

The usefulness of symptom-based screening alone is also limited in this population. The World Health Organization’s four-symptom screening rule—current cough, fever, weight loss, or night sweats—has been widely used to rule out active TB in PLHIV, particularly before initiating preventive treatment. However, its performance varies according to antiretroviral therapy status. In a systematic review and meta-analysis, Hamada et al. (2018) [15] reported that the sensitivity of this rule was lower in PLHIV receiving antiretroviral therapy than in those not receiving treatment, whereas the addition of chest radiography increased sensitivity at the expense of lower specificity. These findings reinforce that, in TB-HIV, the absence of classic symptoms does not exclude active disease, and that clinical screening should be integrated with microbiological, molecular, radiological, or non-sputum-based tests.

One of the main diagnostic obstacles is paucibacillary disease. As CD4+ T-cell counts decline, the amount of *M. tuberculosis* detectable in respiratory secretions is often lower, increasing the probability of negative sputum smears and delaying microbiological confirmation. In previous reviews, the sensitivity of sputum smear microscopy in PLHIV has been considerably lower than that observed with molecular tests. Therefore, a negative sputum smear result should not be used to rule out active TB in patients with high clinical suspicion [7,16].

Another relevant challenge is the difficulty of obtaining adequate samples. Many patients with advanced HIV disease are unable to produce sufficient sputum or present with predominantly extrapulmonary disease, requiring less accessible samples such as pleural fluid, cerebrospinal fluid, lymph node aspirates, biopsies, or blood. These samples may require invasive procedures, specialized infrastructure, and prolonged processing times, limiting their availability in resource-constrained settings and potentially delaying treatment initiation [3,17].

Extrapulmonary and disseminated forms are particularly important because they are associated with greater clinical severity and mortality. In people with advanced HIV disease, TB may manifest as persistent fever, weight loss, anemia, functional decline, sepsis-like syndrome, or neurological involvement, clinical pictures that overlap with other opportunistic infections. This nonspecific presentation complicates the differential diagnosis and requires a high level of clinical suspicion, even when initial sputum tests, chest radiography, or cerebrospinal fluid studies are negative [17,18].

Mycobacterial culture remains fundamental for diagnostic confirmation and drug susceptibility testing, but its turnaround time limits its usefulness as an initial tool in patients with advanced HIV disease or severe disease. Similarly, rapid molecular tests have improved the detection of TB and rifampicin resistance, but they may be limited by sample availability, low bacillary load, cost, and the infrastructure required for implementation. Therefore, the diagnosis of TB-HIV requires combined strategies that integrate molecular tests, non-sputum-based methods, imaging, culture, and clinical judgment [5,8].

Overall, the diagnostic challenges of TB-HIV arise from the interaction between immunosuppression, low bacillary load, atypical clinical presentations, difficulty obtaining samples, and unequal access to diagnostic technologies. This reality justifies the development of flexible, rapid, and clinically risk-based algorithms capable of identifying pulmonary, extrapulmonary, and disseminated TB without relying exclusively on sputum smear microscopy or sputum samples [7,18]. The main diagnostic challenges of TB in PLHIV are summarized in Table 1.

## 4. Conventional Methods: Clinical Role and Limitations in HIV-Associated TB

### 4.1. Sputum Smear Microscopy

Sputum smear microscopy remains rapid, inexpensive, and widely available, but its sensitivity depends on bacillary load and is therefore substantially reduced in PLHIV with paucibacillary disease. In the review by de Faria et al. (2021) [16], sensitivity ranged from 23.0% to 66.7%, with a mean of 43.3%. Consequently, a negative smear should not exclude active TB when clinical suspicion persists or extrapulmonary or disseminated disease is possible [7].

### 4.2. Mycobacterial Culture

Mycobacterial culture remains essential for microbiological confirmation, detection of viable organisms, and drug susceptibility testing, including in specimens with low bacillary load. Its principal limitations are the prolonged turnaround time and dependence on biosafety infrastructure, specialized laboratories, trained personnel, and reliable sample transport. Culture should therefore complement rapid initial testing, particularly in suspected drug resistance, relapse, treatment failure, or extrapulmonary TB, rather than function as the sole initial diagnostic strategy [5,7,18].

### 4.3. Chest Radiography, Clinical Evaluation, and Invasive Procedures

Chest radiography and clinical evaluation remain important components of the initial diagnostic assessment, but their interpretation is more complex in PLHIV. Advanced immunosuppression may be associated with reduced cavitation, diffuse or lower-zone infiltrates, lymphadenopathy, pleural disease, or an apparently normal radiograph, while symptoms may be absent or overlap with other opportunistic infections. Radiography should therefore support diagnosis and prioritize molecular or microbiological testing, but should not independently confirm or exclude TB [2,7].

When non-invasive testing is inconclusive, particularly in sputum-scarce disease, suspected extrapulmonary TB, or intrathoracic lymphadenopathy, invasive procedures may be necessary. Bronchoscopy with bronchoalveolar lavage, transbronchial biopsy, EBUS-TBNA, pleural or lymph-node biopsy, and histopathological examination may improve microbiological or morphological confirmation. Although these methods require specialized expertise and infrastructure, they remain important complementary tools in referral centers and in cases requiring site-directed diagnosis [19,20].

## 5. Rapid Molecular Tests: Clinical Use of Xpert MTB/RIF and Xpert Ultra

### 5.1. Xpert MTB/RIF

In PLHIV, Xpert MTB/RIF provides rapid detection of *M. tuberculosis* and rifampicin resistance with substantially greater sensitivity than sputum smear microscopy. MacLean et al. (2019) [7] reported an approximate sensitivity of 79% and specificity of 98% in this population. Nevertheless, a negative result does not exclude TB when clinical suspicion is high, particularly in paucibacillary, extrapulmonary, or disseminated disease.

### 5.2. Xpert MTB/RIF Ultra

Xpert Ultra improves molecular sensitivity while retaining rapid detection of rifampicin resistance. In the 2025 Cochrane update, pooled sensitivity and specificity for pulmonary TB were 90.7% and 94.8%, respectively. Among PLHIV, sensitivity was 87.7% and specificity was 95.3%, while sensitivity in smear-negative, culture-positive TB was 80.7%. These findings support its use as a preferred initial molecular test, although negative results still require clinical interpretation in paucibacillary disease or when specimen quality is suboptimal [21].

### 5.3. Interpretation and Clinical Follow-Up of Trace-Positive Results

The increased sensitivity of Xpert Ultra requires careful interpretation of the lowest semiquantitative category, reported as “trace detected.” A trace-positive result may represent active paucibacillary TB, residual mycobacterial DNA after previous treatment, or, less commonly, contamination or analytical error. In a community-screening study in Uganda, only 14% of participants with trace-positive sputum were culture-positive at baseline, but 24% met broader microbiological criteria and 26% were classified as having TB after clinical assessment; radiographic abnormalities were also more frequent than among Ultra-negative participants. Therefore, trace positivity should not be automatically interpreted either as definitive active disease or as a false-positive result, and should prompt review of previous TB, symptoms, radiological findings, repeat molecular testing, culture, and clinical evolution [7,21,22].

Longitudinal evidence further suggests that trace positivity may identify early disease with a meaningful risk of progression. Among untreated participants with trace-positive sputum, the cumulative hazard of TB was 24% at one year and 33% at two years, compared with 3% at two years among Ultra-negative controls. An abnormal baseline chest radiograph strongly predicted subsequent TB diagnosis (hazard ratio, 14.6; 95% CI, 3.3–63.8), whereas symptoms alone did not. Although this cohort was not restricted to PLHIV, these findings are particularly relevant to patients with HIV, in whom paucibacillary disease and atypical symptoms are common. Accordingly, trace-positive results warrant prompt reassessment, additional microbiological testing, and close follow-up; treatment should be considered when abnormal chest imaging, advanced immunosuppression, severe illness, or other high-risk features are present [23].

### 5.4. Detection of Rifampicin Resistance

For rifampicin resistance detection, Xpert Ultra also shows high performance. Horne et al. (2025) [21] reported a pooled sensitivity of 95.8% and specificity of 98.3%, with high-certainty evidence. These data support its use as an initial test to identify rifampicin resistance. However, an Xpert Ultra result does not provide a complete resistance profile for other anti-TB drugs; therefore, complementary testing should be considered when drug-resistant TB is suspected or when individualized therapeutic regimens are required.

### 5.5. Clinical and Programmatic Considerations

Xpert Ultra should be interpreted as a high-sensitivity initial test rather than as a definitive rule-out assay. When the result is negative but clinical suspicion remains high, evaluation should continue with culture, imaging, urine LF-LAM, site-directed or extrapulmonary samples, and clinical judgment. Its programmatic impact also depends on cartridge availability, specimen transport, rapid communication of results, access to drug susceptibility testing, and immediate linkage to treatment. Therefore, molecular platforms should be evaluated not only by analytical accuracy, but by their capacity to generate timely and actionable clinical decisions, particularly in people with advanced HIV disease [7,8].

## 6. Urine LAM and FujiLAM in TB-HIV

### 6.1. Biological Rationale for Urine LAM

Urine LAM detection represents one of the most relevant non-sputum-based diagnostic strategies for TB in PLHIV. LAM is a structural glycolipid of the *M. tuberculosis* cell wall that can be released during bacterial replication or degradation and subsequently detected in urine. This characteristic allows the use of a noninvasive, easily obtainable sample with lower biological risk than sputum, which is especially useful in hospitalized, severely ill patients or in those unable to expectorate [7,9].

The usefulness of urine LAM is particularly important in people with advanced HIV disease because this population more frequently presents with extrapulmonary TB, disseminated disease, and paucibacillary forms. In these scenarios, sputum-dependent methods may lose sensitivity because the bacillary load in respiratory secretions is often low or because the patient cannot produce an adequate sample. Detection of the antigen in urine therefore offers a complementary diagnostic pathway that may capture cases that would not be identified through sputum smear microscopy or molecular tests performed on sputum [9,24].

### 6.2. Urine Lateral Flow Lipoarabinomannan Assay: Diagnostic Performance

The lateral-flow urine lipoarabinomannan assay (LF-LAM; Alere Determine TB-LAM Ag or AlereLAM, hereafter AlereLAM) was the first rapid urine-based lateral flow test recommended by the World Health Organization to support the diagnosis of active TB in people with advanced HIV disease. The test can be performed at the point-of-care, does not require complex infrastructure, and provides results in approximately 25 min, giving it an important operational advantage over methods that depend on centralized laboratories [7,9,25].

The Cochrane review by Bjerrum et al. (2019) [9], which included 15 studies in adults living with HIV, showed that LF-LAM has moderate sensitivity but a clearly defined clinical niche. In symptomatic adults, pooled sensitivity was 42% and specificity was 91%; in adults not selected by symptoms, sensitivity was 35% and specificity was 95%.

The diagnostic performance of LF-LAM varies according to the clinical context. In symptomatic adults, sensitivity was higher in inpatients than in outpatients, with values of 52% and 29%, respectively. In adults not selected by symptoms, sensitivity was also higher in inpatients than in outpatients, at 62% versus 31%. This difference confirms that the test is most useful in patients with greater clinical severity, higher pretest probability, and a greater degree of immunosuppression [9].

### 6.3. Evidence of Mortality Impact of LF-LAM in Hospitalized Patients with HIV

In the trial by Peter et al. (2016) [24], overall mortality at eight weeks was 21% in the group assigned to LAM compared with 25% in the group without LAM, representing an absolute reduction of 4% and a relative risk reduction of 17%. The country-adjusted relative risk was 0.83, with statistical significance. These findings demonstrated that a LAM-guided diagnostic strategy can translate into tangible clinical benefits when implemented in hospitalized patients with HIV and suspected TB.

The benefit observed by Peter et al. (2016) [24] is probably explained by the possibility of obtaining an immediate bedside result, which favors early treatment initiation in people with severe disease. The test appears to offer greater usefulness in hospitals with limited diagnostic resources and in patients with advanced immunosuppression, disseminated disease, or inability to produce sputum. These are precisely the scenarios in which TB-HIV often remains undetected until advanced stages. In addition, detection of LAM in urine has been associated with higher mortality risk in patients with TB-HIV, reinforcing its usefulness not only as a diagnostic marker but also as an indicator of advanced disease and poorer prognosis [24,26].

In STAMP, overall mortality at 56 days was 18% in the intervention group and 21% in the standard-of-care group; the overall difference did not reach conventional statistical significance. However, a reduction in mortality was observed in high-risk subgroups, including patients with CD4+ counts below 100 cells/μL, severe anemia, and clinical suspicion of TB at admission. These results suggest that the benefit of urine-based testing is concentrated mainly in patients with a higher probability of advanced or undiagnosed disease [27].

The STAMP study also showed that the greatest incremental benefit of the urine-based strategy came from TB-LAM rather than urine Xpert. In addition, the authors noted that urine-based screening may reduce the risk of hospital discharge with undiagnosed TB, a critical point in hospitalized patients with HIV, in whom early post-discharge mortality may be related to diagnostic delays or lack of timely treatment [27].

### 6.4. FujiLAM and FujiLAM II: Diagnostic Performance and Quality-Control Challenges

Fujifilm SILVAMP TB LAM, subsequently referred to as the original FujiLAM assay, was developed as a next-generation urine lipoarabinomannan test designed to improve sensitivity while preserving the operational advantages of a rapid, point-of-care, non-sputum-based assay. Through the use of high-affinity monoclonal antibodies and silver amplification technology, the assay can detect lower LAM concentrations than LF-LAM, making it particularly relevant to PLHIV with advanced immunosuppression, paucibacillary TB, or disseminated disease [9,10].

In the individual participant data meta-analysis by Broger et al. (2020) [10], the original FujiLAM assay achieved an overall sensitivity of 70.7%, compared with 34.9% for LF-LAM. Among participants with CD4+ counts ≤ 100 cells/μL, sensitivity reached 87.1% for FujiLAM and 56.0% for LF-LAM. These findings established the diagnostic potential of a higher-sensitivity urine LAM assay, particularly in patients with advanced HIV disease.

However, subsequent prospective multicenter evaluations identified clinically important lot-to-lot variability in the original FujiLAM assay, with substantial differences in sensitivity and specificity among manufacturing batches. This limitation affects the interpretation of earlier accuracy estimates: results obtained with individual lots should not be assumed to represent stable performance across all production batches. Consequently, evidence generated with the original assay demonstrates its biological and diagnostic potential but was insufficient to support routine programmatic implementation without improved manufacturing consistency and quality control [28,29].

FujiLAM II was therefore redesigned to address these quality-control limitations. Chikamatsu et al. reported consistent performance across three manufacturing lots in stored urine specimens, whereas Adzemovic et al. observed a sensitivity of 54% and specificity of 95% among outpatients with advanced HIV disease. More recently, Biatu et al. reported an overall sensitivity of 48% and specificity of 96%, with similar positivity across four manufacturing lots and higher sensitivity than AlereLAM. These findings support improved inter-lot precision, although the variability in sensitivity across studies highlights the influence of clinical setting, degree of immunosuppression, specimen characteristics, and the reference standard used [30,31,32].

In an updated systematic review and meta-analysis including 13 studies and 5819 participants, pooled sensitivity and specificity for FujiLAM assays were 63% and 90%, respectively. Sensitivity was higher among inpatients and participants with lower CD4+ counts. In subgroup analyses, estimated sensitivity was 65% for the original FujiLAM assay and 56% for FujiLAM II, with overlapping confidence intervals. Therefore, current evidence supports improved manufacturing consistency for FujiLAM II but does not establish that it is uniformly more sensitive than the original assay [33].

FujiLAM II should consequently be considered a promising emerging technology rather than a replacement for currently recommended diagnostic tests. Additional prospective evaluations using fresh specimens, broader geographical and clinical populations, formal quality-assurance systems, cost-effectiveness analyses, and studies of clinical impact are required before widespread programmatic adoption. Until such evidence and formal guideline assessment are available, LF-LAM should remain the urine LAM assay incorporated into current WHO-recommended diagnostic algorithms, while FujiLAM II may be considered in research or specifically validated implementation settings [5,30,31,32,33].

### 6.5. Integration of LAM with Xpert Ultra in Diagnostic Algorithms

The accumulated evidence indicates that no single test is sufficient to cover the entire clinical spectrum of TB-HIV. Xpert Ultra offers high sensitivity in respiratory samples and detects rifampicin resistance, but it may fail when sputum cannot be obtained or when disease is extrapulmonary or disseminated. Urine LF-LAM provides a currently recommended non-sputum-based option in eligible PLHIV, whereas FujiLAM II remains an emerging assay under evaluation. Neither urine LAM assay provides information on drug resistance, and diagnostic performance varies according to the degree of immunosuppression and clinical context [5,8,9,30,31,32,33].

The integration of sputum- and urine-based tests makes it possible to take advantage of complementary diagnostic mechanisms. Xpert Ultra detects *M. tuberculosis* DNA in respiratory samples with high sensitivity, whereas urine LAM may reflect disseminated disease or greater systemic antigenic burden, particularly in people with advanced HIV disease. This complementarity explains why combined algorithms can increase overall diagnostic yield compared with use of a single test alone [18,34].

In hospitalized patients with HIV, the most reasonable approach should prioritize rapid testing from admission. In patients with compatible symptoms, signs of severity, low CD4+ counts, anemia, weight loss, persistent fever, or inability to expectorate, simultaneous use of Xpert Ultra in sputum—when available—and urine LAM could reduce diagnostic delays and favor early treatment initiation. This strategy is consistent with evidence of reduced mortality observed in high-risk hospitalized patients and with evidence that symptom-based algorithms alone are insufficient in hospitalized patients with HIV [24,27,35].

In outpatients, LAM use should be more selective, given that it may have different implications in settings with lower TB prevalence. In this group, Xpert Ultra remains a priority initial test when PTB is suspected. However, in people with advanced HIV disease, systemic symptoms, difficulty producing sputum, or high suspicion of extrapulmonary disease, LAM may provide additional useful information for deciding on treatment or continuing diagnostic evaluation [7,9].

A critical aspect of combined algorithms is that negative results should not be interpreted in isolation. A negative Xpert Ultra result does not exclude TB in patients with advanced HIV disease, especially if an adequate sample was not obtained or if extrapulmonary disease is present. Similarly, a negative LF-LAM result does not rule out TB because of its moderate sensitivity. Therefore, clinical decisions should integrate pretest probability, disease severity, specimen availability, imaging, culture, and, when necessary, empirical treatment in scenarios of high suspicion [7,8].

From a programmatic perspective, incorporating LAM and Xpert Ultra into integrated algorithms could have special impact in low- and middle-income countries, where TB-HIV coinfection continues to be associated with diagnostic delays, hospitalization, and preventable mortality. To maximize their usefulness, these tests must be accompanied by clear pathways for treatment initiation, linkage to care, resistance evaluation, and clinical follow-up. Available evidence suggests that diagnostic benefit translates into better outcomes only when the result generates timely clinical action [18,24,27].

The main established diagnostic tools for TB in PLHIV, including conventional methods, rapid molecular tests, and urine-based assays, are summarized in Table 2.

## 7. Host Blood Biomarkers for TB Diagnosis and Screening in People Living with HIV

### 7.1. Rationale and Current Diagnostic Role

Blood biomarkers are being investigated as accessible, non-sputum-based tools for TB screening and diagnostic prioritization. The World Health Organization target product profiles recognize that different tests may function as high-sensitivity rule-out tools, components of two-step screening strategies, or methods for prioritizing confirmatory evaluation. Blood-based platforms are attractive because they could be adapted to rapid, low-cost, point-of-care formats using venous or capillary samples [11,36].

Their main limitation in TB-HIV is reduced specificity. Advanced HIV disease may be accompanied by opportunistic infections, bacterial or fungal disease, malignancy, malnutrition, and chronic immune activation, all of which can modify inflammatory biomarkers. Consequently, host-response tests should be interpreted primarily as triage or initial rule-out tools rather than independent confirmation of active TB [11,37].

### 7.2. C-Reactive Protein

C-reactive protein (CRP) is the most extensively evaluated blood biomarker for TB screening. In the meta-analysis by Gaeddert et al. (2024) [11], a threshold of 10 mg/L provided pooled sensitivity of 86% and specificity of 67% for pulmonary TB. Among PLHIV, pooled sensitivity increased to 93%, whereas specificity decreased to 59%, a pattern consistent with a sensitive but nonspecific triage test.

Primary studies in Uganda and South Africa also found that CRP had sensitivity comparable to symptom screening but greater specificity in some outpatient populations. Therefore, CRP may help identify patients who require Xpert Ultra, culture, urine LF-LAM, or radiological evaluation, but an elevated result should not independently confirm TB or determine treatment initiation [11,38,39].

### 7.3. Other Protein Biomarkers and Host-Response Signatures

IP-10, NCAM-1, and serum amyloid A have also been evaluated as candidate biomarkers. A systematic review reported pooled sensitivity and specificity of 85% and 63% for IP-10, while evidence for NCAM-1 and serum amyloid A remains heterogeneous across populations, platforms, and cut-off points. HIV-associated immune activation and concurrent infections may further reduce specificity; these markers should therefore remain investigational until prospectively validated in populations stratified by CD4+ count, antiretroviral therapy status, viral suppression, and coinfections [11,37].

Multiprotein and transcriptomic signatures may improve performance by combining several components of the host response. Mulenga et al. (2020) [12] identified 25 transcriptomic signatures evaluated across 68 cohorts, several of which met at least one minimum WHO target product profile criterion. Nevertheless, molecular complexity, cost, limited point-of-care availability, and insufficient validation in advanced HIV disease restrict their current clinical applicability. HIV and opportunistic infections may also produce gene-expression patterns that overlap with active TB [11].

### 7.4. Clinical Applicability

The most plausible current role of host biomarkers in PLHIV is to prioritize confirmatory evaluation, particularly in outpatient or high-volume settings where molecular testing capacity is limited. They may also contribute when sputum cannot be obtained or when integrated with Xpert Ultra, urine LF-LAM, chest radiography, and clinical assessment. However, positive results require molecular, microbiological, imaging, or site-directed confirmation, and none of the emerging protein or transcriptomic signatures is currently suitable as a stand-alone diagnostic test [11,12,18,40].

## 8. Artificial Intelligence and Computer-Aided Chest Radiography in HIV-Associated Tuberculosis

### 8.1. Role and Overall Diagnostic Performance

Computer-aided detection (CAD) systems analyze digital chest radiographs and generate a probability score for pulmonary TB. Their principal role is screening, triage, or support for the initial diagnostic evaluation rather than stand-alone confirmation. They may be particularly useful in active case-finding programs, primary care, prisons, shelters, migrant populations, and settings where expert radiological interpretation is limited [13,14,41].

In an individual participant data meta-analysis including 3727 participants, Tavaziva et al. (2022) [13] found broadly comparable performance for CAD4TB, Lunit, and qXR. At a fixed sensitivity of approximately 90%, specificity was 56.9%, 54.1%, and 60.5%, respectively. Gelaw et al. (2023) [14] similarly reported moderate-to-good discrimination, with areas under the curve of 0.85 for Lunit, 0.75 for qXR, and 0.71 for CAD4TB. These findings support the use of CAD to prioritize molecular or microbiological testing, but not to establish or exclude TB independently.

### 8.2. Performance and Limitations in People Living with HIV

CAD performance may be lower in PLHIV and in smear-negative disease. Tavaziva et al. reported adjusted reductions in sensitivity of approximately 13 percentage points for CAD4TB and qXR in people with HIV, whereas Lunit did not show a statistically significant reduction. Across the evaluated systems, sensitivity was also approximately 12–17 percentage points lower in smear-negative than in smear-positive TB. These limitations are clinically relevant because advanced HIV disease may produce less cavitation, lymphadenopathy, diffuse or interstitial abnormalities, and other subtle or atypical radiographic patterns [7,13].

A negative CAD result should therefore not rule out TB when clinical suspicion remains high, particularly in patients with low CD4+ counts, systemic deterioration, extrapulmonary involvement, or inability to produce sputum. Because CAD systems generate continuous probability scores, diagnostic thresholds should be locally validated according to TB prevalence, available confirmatory capacity, HIV status, and the consequences of false-negative and false-positive results [13,41].

### 8.3. Integration into Diagnostic Pathways

CAD may reduce dependence on expert readers and has shown diagnostic accuracy comparable to human radiographic interpretation in some evaluations. However, its clinical value depends on the availability of a clear pathway for Xpert Ultra, culture, urine LF-LAM, or additional evaluation after a positive or clinically discordant result. In community and primary care programs, it may support high-volume screening and diagnostic prioritization; in hospitalized patients with advanced HIV disease, it should be interpreted only as one component of a broader, parallel diagnostic evaluation [7,8,13,14].

Accordingly, CAD should complement rather than replace clinical assessment and microbiological or molecular confirmation. Its real-world impact will depend on locally validated thresholds, quality assurance, access to confirmatory testing, and timely linkage to treatment. The principal emerging triage and screening tools for TB in PLHIV are summarized in Table 3.

## 9. Extrapulmonary, Disseminated, and Severe Forms of TB in People Living with HIV

### 9.1. Clinical Importance of Extrapulmonary TB in TB-HIV

Extrapulmonary TB represents one of the greatest diagnostic challenges in PLHIV. Unlike PTB, which is usually evaluated using respiratory samples, extrapulmonary TB may involve lymph nodes, pleura, the central nervous system, bone, the genitourinary tract, peritoneum, skin, and other organs. This anatomical diversity complicates clinical suspicion, delays sample collection, and reduces the performance of conventional sputum-based tests [7,42].

In people with HIV, immunosuppression modifies the clinical presentation of TB and favors extrapulmonary and disseminated forms, particularly as CD4+ T-cell counts decline. Consequently, paucibacillary disease, nonspecific systemic manifestations, atypical radiographic findings, and involvement of multiple anatomical sites become increasingly common, reducing the diagnostic yield of approaches based primarily on respiratory samples [7,18].

The importance of extrapulmonary TB lies not only in its frequency but also in its clinical impact. Some forms, such as tuberculous meningitis, disseminated TB, and *M. tuberculosis* bacteremia, are associated with high mortality, especially when diagnosis is delayed. This requires broader diagnostic strategies that combine clinical assessment, molecular tests, urine-based tests, imaging, culture, and targeted collection of extrapulmonary samples [17,18].

### 9.2. Challenges in Obtaining Extrapulmonary Samples

The diagnosis of extrapulmonary TB often requires samples from the affected site, such as lymph node biopsies, pleural fluid, cerebrospinal fluid, bone tissue, peritoneal fluid, or other invasive samples. Obtaining these samples may be technically difficult, costly, painful, or unavailable in primary care centers. In addition, many of these specimens contain low bacillary loads, which decreases the sensitivity of sputum smear microscopy, culture, and some molecular tests [42].

Low bacterial load is one of the main diagnostic obstacles. In extrapulmonary TB, the presence of *M. tuberculosis* may be focal, scarce, or irregularly distributed within the affected tissue. Therefore, even a correctly obtained sample may be negative if it does not contain enough bacilli. This situation is especially problematic in people with HIV, in whom disease may be disseminated but microbiological yield from conventional samples may remain low [7,9].

Culture remains essential for microbiological confirmation, species identification, and drug susceptibility testing. However, its prolonged turnaround time limits its usefulness for initial decision-making in patients with advanced HIV disease or severe disease, reinforcing the need to combine culture with rapid molecular and non-sputum-based diagnostic approaches [8,17].

### 9.3. Disseminated Tuberculosis and Mycobacterium tuberculosis Bacteremia

Disseminated TB represents the severe end of the disease spectrum in people with HIV. In this context, *M. tuberculosis* may spread beyond the lung and affect multiple organs, with nonspecific clinical manifestations such as persistent fever, weight loss, anemia, functional decline, sepsis-like syndrome, or organ failure. These manifestations overlap with other opportunistic infections, complicating the differential diagnosis [18].

*M. tuberculosis* bacteremia is a frequent and clinically relevant manifestation of disseminated TB in people with advanced HIV disease. In an individual participant data meta-analysis, Barr et al. (2020) [43] estimated that the probability of *M. tuberculosis* bloodstream infection among hospitalized patients with TB-HIV, World Health Organization danger signs, and a median CD4+ count of 76 cells/μL was 45%. In addition, bacteremia was associated with a higher risk of death before 30 days and delaying anti-TB treatment initiation for more than four days increased mortality. These findings explain why disseminated TB in advanced HIV disease should be approached as a diagnostic and therapeutic emergency [18].

*M. tuberculosis* bacteremia illustrates why sputum-dependent tests may be insufficient in patients with advanced HIV disease. A patient may have severe systemic disease without a positive respiratory sample or even without the ability to expectorate. This justifies the development and evaluation of tests using non-respiratory samples, such as urine, blood, stool, or oral swabs, as well as the use of host biomarkers and clinical algorithms that do not rely exclusively on sputum [7,18].

### 9.4. Central Nervous System Tuberculosis

Central nervous system TB is one of the most severe extrapulmonary forms. It may manifest as tuberculous meningitis, tuberculomas, tuberculous abscesses, or other less frequent forms. Although it represents a smaller proportion of total TB cases, it is associated with high mortality and important neurological sequelae, even when appropriate anti-TB treatment is administered [17].

In people with HIV, the risk of central nervous system TB increases significantly. Nelson and Zunt (2011) [17] note that people with TB-HIV are more likely to present with central nervous system involvement than people without HIV. In addition, the risk appears to increase in patients with CD4+ counts below 100 cells/μL, reinforcing the need to maintain high clinical suspicion in individuals with advanced immunosuppression and neurological symptoms.

The clinical presentation of tuberculous meningitis may be incomplete or atypical in people with HIV. Although classic symptoms include fever, headache, vomiting, altered mental status, and meningeal signs, some studies have shown that only a minority of patients with HIV present with the classic triad of fever, headache, and meningeal signs. This variability may delay diagnosis, especially when other opportunistic infections coexist, such as cryptococcosis or cerebral toxoplasmosis [17].

### 9.5. Diagnostic Limitations in Cerebrospinal Fluid

The diagnosis of central nervous system TB depends largely on cerebrospinal fluid analysis. However, available tests have important limitations. Cerebrospinal fluid smear microscopy has low sensitivity, and culture, although specific, may take weeks. Molecular tests have improved initial diagnosis: in a prospective cohort of adults with suspected meningitis in Uganda, Cresswell et al. (2020) [44] found that Xpert Ultra performed on cerebrospinal fluid had higher sensitivity than Xpert MTB/RIF and MGIT culture in a population with a high burden of HIV. Nevertheless, even Xpert Ultra showed imperfect negative predictive value; therefore, a negative result should not be used to rule out tuberculous meningitis when clinical suspicion is high [17].

In people with advanced HIV disease, cerebrospinal fluid findings may be less typical. Nelson and Zunt (2011) [17] describe that some patients with HIV and tuberculous meningitis may not present pleocytosis and that cell counts may be lower among those with very low CD4+ counts. This reduces the usefulness of classic cerebrospinal fluid patterns and requires interpretation of results within the patient’s clinical and immunological context.

Because no cerebrospinal fluid test has perfect sensitivity, empirical treatment may be necessary when clinical suspicion of tuberculous meningitis is high. This decision is especially relevant in patients with HIV, in whom therapeutic delay may increase the risk of death or irreversible neurological damage. Therefore, diagnostic tests should support, but not replace, clinical judgment in severe forms of extrapulmonary TB [17].

### 9.6. Non-Respiratory Samples and Emerging Diagnostic Directions

The difficulty of obtaining adequate sputum or extrapulmonary samples has driven the study of diagnostic tests using non-respiratory specimens. Among the most relevant alternatives are urine, blood, stool, and oral or nasopharyngeal swabs. These samples may be more accessible and less invasive, especially in patients with advanced HIV disease, disseminated disease, or inability to expectorate [18].

Urine-based LAM is especially important because it supports TB detection without depending exclusively on the anatomical site of disease. LF-LAM has an established role in eligible PLHIV, whereas FujiLAM II remains an emerging assay under evaluation. Diagnostic yield is generally higher in hospitalized patients and those with low CD4+ counts, precisely the groups at highest risk of disseminated TB and early mortality. In this sense, urine represents a strategic specimen for closing the diagnostic gap in people with advanced HIV disease [5,9,10,30,31,32,33].

Molecular tests in blood and urine have also been explored, particularly Xpert Ultra applied to non-respiratory samples. Although these approaches are promising, they still require additional validation, especially to determine their sensitivity, specificity, clinical usefulness, and programmatic feasibility. Current evidence suggests that these tests could be more useful in disseminated disease or hospitalized patients, but they do not yet replace established diagnostic algorithms [18].

In extrapulmonary TB, fluid biomarkers may provide supportive evidence when direct microbiological confirmation is difficult. For pleural TB, meta-analyses have reported pooled sensitivity and specificity of 92% and 90%, respectively, for adenosine deaminase (ADA), and 93% and 96% for unstimulated interferon-gamma (IFN-γ). Unstimulated cerebrospinal fluid IFN-γ has also shown pooled sensitivity of 86% and specificity of 92% for tuberculous meningitis. Guideline-based evidence supports ADA measurement in pleural, peritoneal, pericardial, and cerebrospinal fluids and free IFN-γ measurement in pleural and peritoneal fluids; however, these are conditional recommendations based on low-quality evidence. Thresholds vary, study risk of bias is substantial, and data specific to PLHIV remain limited. Therefore, neither biomarker should be used as a stand-alone test; their greatest value lies in complementing Xpert MTB/RIF Ultra, culture, imaging, and site-directed specimen collection within anatomically adapted diagnostic algorithms [45,46,47,48].

### 9.7. Implications for Diagnostic Algorithms in Advanced HIV Disease

Extrapulmonary and disseminated TB in people with advanced HIV disease requires reconsideration of diagnostic algorithms centered exclusively on sputum. In these patients, the absence of an adequate respiratory sample or a negative sputum result does not exclude active disease, because the primary site of infection may be outside the lung, or the respiratory bacillary load may be low. Therefore, diagnostic evaluation should consider from the outset the possibility of systemic disease, extrapulmonary involvement, and the need for alternative samples according to the clinically suspected site [7,18].

In this context, negative results should be interpreted with caution. A negative molecular test in sputum may reflect the absence of bacilli in the sample rather than the absence of TB; a negative LAM test does not rule out disease because of its moderate sensitivity; and a chest radiograph may be normal or atypical in advanced immunosuppression. Therefore, decision-making should integrate clinical severity, CD4+ count, systemic symptoms, radiological findings, availability of extrapulmonary samples, culture, and patient evolution [7,9,18].

The main clinical implication is that, in advanced HIV disease, diagnosis should be guided by risk and severity rather than by a rigid sequence of tests. When there is high suspicion of extrapulmonary or disseminated TB, evaluation should be expanded early to non-respiratory samples, urine-based tests, targeted imaging, and culture, without delaying therapeutic decisions in severely ill patients. Formal integration of these tools is developed in the following section, which focuses on integrated diagnostic algorithms and future perspectives [17,18].

In summary, extrapulmonary, disseminated, and central nervous system TB represent one of the main reasons why TB-HIV diagnosis remains complex. Low bacillary load, difficulty obtaining samples, nonspecific clinical presentation, and high mortality require flexible, rapid diagnostic strategies centered on clinical risk. For this population, the combination of molecular tests, urine LAM, extrapulmonary samples, culture, imaging, and clinical judgment remains indispensable, while future perspectives for diagnostic integration are discussed in the next section [7,17,18].

## 10. Discussion: Integrated Diagnostic Algorithms

### 10.1. No Single Test Resolves TB-HIV Diagnosis: Hierarchy of Evidence and Clinical Use

The diagnosis of TB in PLHIV requires an integrated approach because no single test adequately covers the full clinical spectrum of disease. Xpert Ultra offers high sensitivity for PTB and rapid detection of rifampicin resistance, but it depends on the availability of adequate respiratory or extrapulmonary samples. Urine LF-LAM provides a currently recommended non-sputum-based option in eligible PLHIV, whereas FujiLAM II remains an emerging assay under evaluation; neither provides information on drug resistance, and diagnostic performance varies according to the degree of immunosuppression and clinical context [5,8,9,11,13,30,31,32,33].

Because TB in PLHIV encompasses pulmonary, extrapulmonary, disseminated, paucibacillary, and asymptomatic presentations, diagnostic algorithms should be individualized according to disease presentation, degree of immunosuppression, specimen availability, and illness severity. Hospitalized patients with advanced HIV disease require rapid diagnostic strategies aimed at reducing mortality, whereas outpatient evaluation should balance diagnostic accuracy, resource utilization, and timely microbiological confirmation [7,18].

From this perspective, TB-HIV diagnosis should be understood as a dynamic clinical decision-making process rather than the result of a single diagnostic test. The value of each diagnostic technology depends not only on its analytical performance, but also on its availability, turnaround time, feasibility in the local setting, capacity to influence therapeutic decisions, and integration into patient-centered diagnostic pathways. In advanced HIV disease, where diagnostic delay rapidly translates into increased mortality, combining complementary diagnostic tools according to clinical presentation is more important than maximizing the performance of any individual assay [7,24,27].

Accordingly, the technologies reviewed can be organized according to guideline status, strength of clinical evidence, intended diagnostic role, and readiness for implementation rather than through a single ranking based exclusively on diagnostic accuracy. The first level comprises core diagnostic tools with consolidated evidence or current guideline support: rapid molecular testing, preferably Xpert MTB/RIF Ultra; urine LF-LAM in eligible people living with HIV; mycobacterial culture and drug susceptibility testing; site-directed specimen collection; and chest radiography as an initial imaging or triage tool rather than as stand-alone confirmation. The second level includes established, context-dependent complementary methods, such as sputum smear microscopy, histopathology, bronchoscopy, EBUS-TBNA, mycobacterial blood culture, ADA, and unstimulated IFN-γ, which become particularly important when routine samples are unavailable or extrapulmonary disease is suspected. CRP and computer-aided chest radiography occupy an intermediate triage role because they may prioritize confirmatory testing when locally validated but cannot establish a definitive diagnosis. The third level comprises emerging or investigational approaches, including FujiLAM II, multiprotein and transcriptomic signatures, and molecular testing of alternative specimens outside currently validated indications. These technologies should complement, but not replace, the core diagnostic pathway until additional prospective, operational, and clinical-impact evidence becomes available [5,8,9,11,13,17,18,30,31,32,33].

### 10.2. Algorithm for Hospitalized Patients with Advanced HIV Disease

Hospitalized patients with advanced HIV disease represent the group of highest diagnostic priority because of their elevated mortality, higher frequency of disseminated TB, and high probability of being unable to produce sputum. In this scenario, the initial approach should ideally include, from admission, targeted clinical assessment, systematic symptom screening, chest radiography, Xpert Ultra in sputum if the patient can expectorate, urine LF-LAM, culture when available, and collection of extrapulmonary samples when there is suspicion of a specific anatomical site. This approach is aligned with evidence showing that strategies based only on clinical screening have important limitations in hospitalized patients with HIV and that broader use of rapid molecular testing may be more appropriate in hospital settings with high TB prevalence [5,8,9,18,35].

In this group, urine LAM has a particularly important role because it can be performed rapidly, at the bedside, and without depending on respiratory samples. The trial by Peter et al. (2016) [24] demonstrated that a LAM-guided strategy in hospitalized patients with HIV and suspected TB reduced mortality at eight weeks, with an absolute reduction of 4% and a relative risk reduction of 17%. This finding is fundamental because it shows that a diagnostic test can modify clinical outcomes when integrated promptly into decision-making.

This approach is also consistent with the 2025 World Health Organization diagnostic guidance, which supports concurrent testing of respiratory and non-respiratory samples for the initial detection of TB and rifampicin resistance among adults and adolescents living with HIV. In practice, this recommendation reinforces the need to avoid a strictly sequential diagnostic pathway in high-risk patients and supports parallel testing when sputum availability, extrapulmonary involvement, or advanced immunosuppression may reduce the yield of a single specimen or test [5].

The STAMP trial also supports the use of urine-based tests in hospitalized patients with HIV, particularly in high-risk subgroups. Although the intervention did not significantly reduce overall mortality in the entire study population, it showed benefits in patients with CD4+ counts below 100 cells/μL, severe anemia, or clinical suspicion of TB at admission. In addition, the study suggested that urine-based screening may decrease the risk of hospital discharge with undiagnosed TB, a critical situation in people with advanced HIV disease [27].

In hospitalized patients, a practical algorithm should avoid excessive sequential waiting. Instead of requesting one test and waiting for its result before proceeding to the next, complementary tests should preferably be applied in parallel when clinical suspicion is high. For example, a patient with advanced HIV disease, fever, weight loss, anemia, or functional decline should simultaneously receive Xpert Ultra if sputum is obtained, LF-LAM in urine, and chest radiography, together with culture and extrapulmonary samples according to the clinical picture. This strategy increases the probability of early diagnosis and reduces dependence on a single sample or technology [7,9,18].

### 10.3. Algorithm for Outpatients with HIV

In outpatients with HIV, the diagnostic algorithm should balance sensitivity, specificity, accessibility, and cost. In this context, evaluation may begin with systematic symptom screening, CRP measurement when available, chest radiography or CAD in screening programs, and Xpert Ultra as the initial molecular test when PTB is suspected. Unlike the severely ill hospitalized patient, pretest probability may be lower, so tests with moderate specificity should be interpreted with greater caution. However, when Xpert MTB/RIF or Xpert Ultra are used as screening tools regardless of symptoms, their sensitivity in people with HIV may be insufficient to rule out TB in isolation, although specificity remains high; therefore, negative results should be interpreted together with symptoms, CRP, radiography, urine LAM, and clinical probability [7,11,13,49].

CRP may be useful as a triage tool in outpatients with HIV, especially when molecular resources are limited. Its relatively high sensitivity in people with HIV makes it possible to identify patients who require confirmatory testing, although its low specificity prevents its use as a definitive diagnostic test. In this model, elevated CRP does not confirm TB, but it may prioritize Xpert Ultra, culture, or chest radiography; conversely, low CRP could reduce the probability of active disease in certain clinical contexts [11,37].

In outpatients, LF-LAM should be used more selectively than in hospitalized patients. The Cochrane review by Bjerrum et al. (2019) [9] showed that LF-LAM sensitivity is lower in outpatients than in inpatients, so its greatest usefulness is concentrated in people with advanced HIV disease, low CD4+ counts, signs of severity, intense systemic symptoms, or suspected extrapulmonary TB. In outpatients with a lower clinical probability of TB, a positive result should be interpreted with caution and preferably confirmed with molecular or microbiological tests.

Chest radiography, with or without artificial intelligence assistance, may be useful in active case-finding or outpatient screening programs. However, in people with HIV, a normal radiograph or low CAD score should not rule out TB if clinical suspicion, advanced immunosuppression, or persistent systemic symptoms are present. In this group, radiography should function as a prioritization tool, not as a substitute for Xpert Ultra, culture, or urine-based tests when indicated [7,13].

### 10.4. Algorithm for Suspected Extrapulmonary or Disseminated TB

Suspected extrapulmonary or disseminated TB requires an algorithm different from that used for classic PTB. In these cases, diagnosis should not depend exclusively on sputum because the main disease site may be outside the lung or because the patient may have systemic dissemination with low respiratory bacillary load. The approach should include samples from the affected site whenever possible, such as lymph node aspirate, pleural fluid, cerebrospinal fluid, peritoneal fluid, biopsy, or tissue, in addition to culture, histopathology, and molecular tests when available [17,42].

In a preliminary systematic review and meta-analysis, Zhang et al. (2020) [50] reported a high pooled sensitivity of 85.1% and specificity of 97.5% for Xpert Ultra in extrapulmonary TB, although the authors noted heterogeneity by sample type and the need for more specific studies. Therefore, a negative result should not exclude TB when clinical suspicion is high. This is particularly important in tuberculous meningitis, where the sensitivity of cerebrospinal fluid tests may be limited, and therapeutic delay is associated with high mortality and neurological sequelae [8,17].

More recent Cochrane evidence supports the usefulness of Xpert Ultra for extrapulmonary TB, while emphasizing that diagnostic accuracy varies substantially according to specimen type and anatomical site. Xpert Ultra appears particularly useful for lymph node specimens and cerebrospinal fluid; however, in tuberculous meningitis, increased sensitivity may be accompanied by lower specificity compared with Xpert MTB/RIF. Therefore, positive results may support rapid treatment initiation, but negative results should not be used to exclude extrapulmonary or central nervous system TB when clinical suspicion remains high [51].

In disseminated disease or suspected *M. tuberculosis* bacteremia, urine-based tests and non-respiratory samples become more relevant. Sossen et al. (2025) [18] note that, among hospitalized people with TB-HIV, an important proportion may present with *M. tuberculosis* bloodstream infection, associated with sepsis, organ dysfunction, and early mortality. In these patients, LF-LAM, mycobacterial blood culture when available, and molecular tests in alternative samples may complement the initial evaluation.

An algorithm for extrapulmonary or disseminated TB should begin with three clinical questions: first, is there an anatomical site accessible for sample collection? Second, are there signs of severe systemic disease that justify non-sputum-based testing and early treatment? Third, is there a risk of drug resistance that requires expanded susceptibility testing? This logic allows the approach to be adapted to the clinical presentation, preventing the absence of sputum or a negative test from delaying treatment in high-risk patients [7,18].

### 10.5. Diagnosis of Drug Resistance

Early detection of drug resistance is an essential component of any diagnostic algorithm in TB-HIV. Xpert MTB/RIF and Xpert Ultra can identify rifampicin resistance at the same time as they detect *M. tuberculosis*, favoring timely initiation of appropriate regimens in patients with drug-resistant TB. In the Cochrane review by Zifodya et al. (2021), Xpert Ultra and Xpert MTB/RIF showed comparable performance for rifampicin resistance, with sensitivities close to 95% and specificities close to 99% [8].

However, rifampicin resistance does not exhaust the problem of drug resistance. People with HIV may have TB resistant to isoniazid, fluoroquinolones, or other relevant drugs, and Xpert Ultra does not provide complete information on these profiles. In this context, Xpert MTB/XDR represents a relevant complementary tool because it can detect mutations associated with resistance to isoniazid, fluoroquinolones, ethionamide, and second-line injectable drugs in patients with confirmed pulmonary TB. Nevertheless, its usefulness should be interpreted within the current therapeutic landscape, because it does not detect resistance to key drugs used in more recent regimens, such as bedaquiline or linezolid. Therefore, when rifampicin resistance is detected or when there is high clinical or epidemiological suspicion of drug-resistant TB, additional tests should be considered, such as Xpert MTB/XDR, line probe assays, culture with phenotypic drug susceptibility testing, or targeted sequencing when available [5,8,18,52].

The challenge is greater in patients with advanced HIV disease who cannot produce sputum or who present with extrapulmonary TB. In these cases, confirmation of resistance may be delayed by the difficulty of obtaining adequate samples. Therefore, a future priority is to validate rapid resistance tests in non-respiratory samples, such as cerebrospinal fluid, lymph node aspirates, urine, blood, or tissue. This need is particularly important in severe forms such as tuberculous meningitis, disseminated disease, and TB in hospitalized patients [17,18].

### 10.6. Implementation and Economic Considerations in Low- and Middle-Income Countries

Implementation of integrated diagnostic algorithms depends on health-system capacity. In low- and middle-income countries, limitations in cartridge supply, equipment maintenance, laboratory infrastructure, trained personnel, and sample transport may reduce the real-world impact of otherwise highly accurate diagnostic tests [5,7,18,53].

Although the expansion of antiretroviral therapy has substantially reduced HIV-related morbidity and the frequency of severe presentations, it does not eliminate the relevance of diagnostic algorithms for advanced HIV disease. In 2024, ART coverage reached 91% among people with diagnosed and notified TB-HIV, but only 61% when assessed against the estimated total number of incident TB-HIV cases, indicating that a substantial proportion remained outside timely diagnosis and treatment. Advanced HIV disease also persists because of late HIV diagnosis, disengagement from care, treatment interruption, and virological failure. Moreover, recent HIV service disruptions associated with suspensions or reductions in official development assistance have affected diagnostic monitoring and ART delivery in some settings. These vulnerabilities could increase delayed or advanced presentations and reinforce the need to maintain rapid, parallel TB diagnostic pathways for severely ill patients and those with advanced HIV disease [1,4,54].

Although region-specific evidence remains limited, local studies can provide useful information for adapting diagnostic algorithms to concrete contexts. In Chihuahua, Mexico, TB-HIV coinfection showed a heterogeneous distribution according to sociodemographic and geographic variables, supporting the need for diagnostic strategies sensitive to territorial and population contexts [55]. In these scenarios, simple, rapid, non-sputum-based tests—particularly LF-LAM, and potentially FujiLAM II after further prospective validation and programmatic assessment—could have particular value if accompanied by clear pathways for referral, diagnostic confirmation, and timely treatment.

Successful implementation requires more than test availability and should include context-specific economic evaluation. In a South African decision-analytic model, adding the original FujiLAM assay to Xpert Ultra increased projected early treatment and reduced TB-related mortality, with an incremental cost-effectiveness ratio of USD 676.9 per disability-adjusted life-year averted. However, the analysis relied on assumed FujiLAM costs and diagnostic performance from the original assay, so its conclusions cannot be directly extrapolated to FujiLAM II. For artificial intelligence-assisted chest radiography, a model from Karachi projected cost reductions of 19–37% and 3–4% more disability-adjusted life-years averted when computer-aided detection was used for triage before smear microscopy or Xpert. Conversely, the PROSPECT randomized study in Malawi found faster TB treatment initiation but no cost-effectiveness for the computer-aided radiographic strategy during its 56-day analytical horizon. These contrasting findings indicate that economic value depends on TB prevalence, software and test prices, diagnostic thresholds, confirmatory-testing capacity, quality assurance, and linkage to treatment. Additional real-world evaluations in PLHIV remain necessary, particularly for FujiLAM II and contemporary computer-aided detection systems [56,57,58].

**Figure 1:**
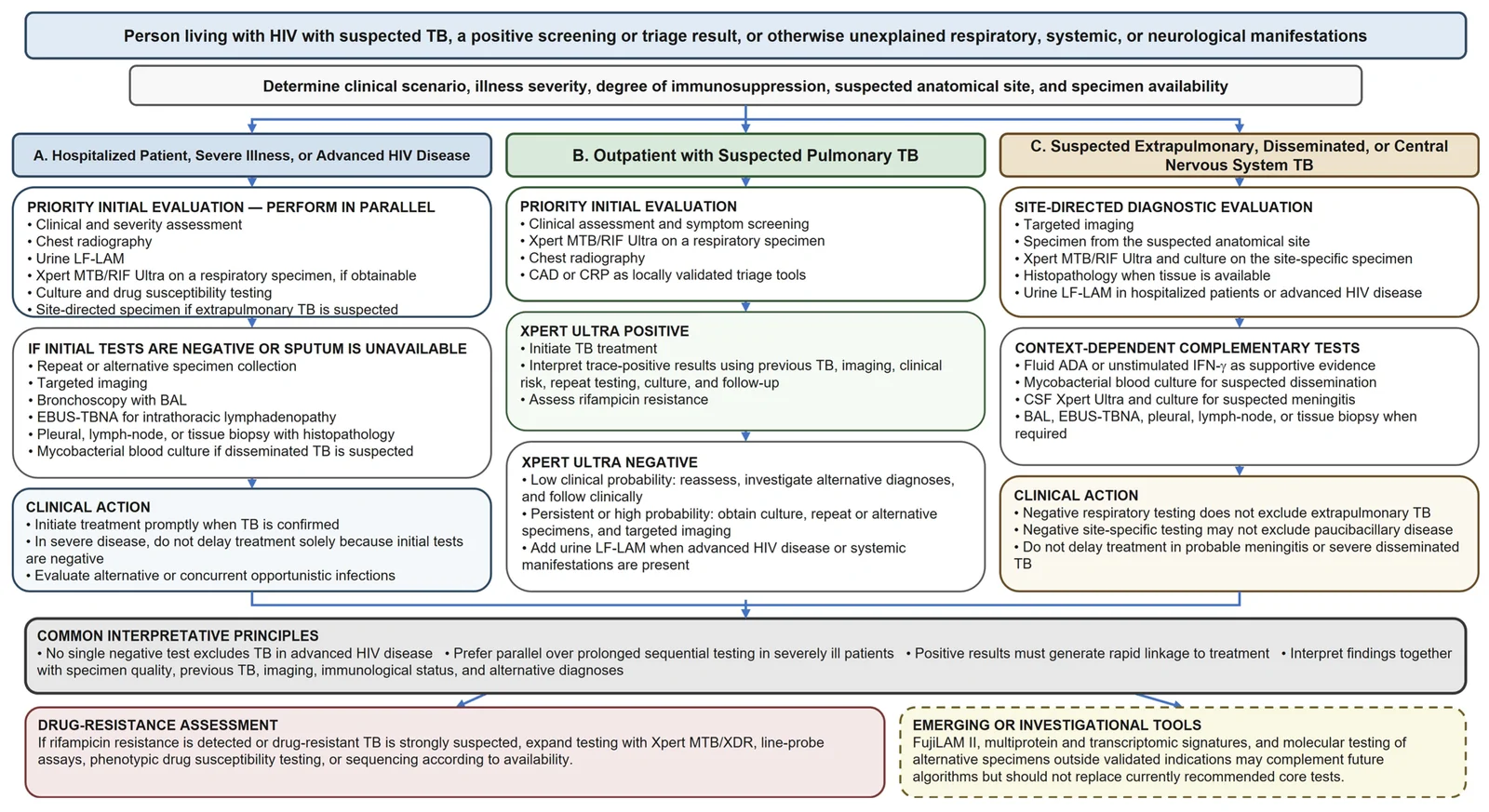
Scenario-based integrated diagnostic pathway for tuberculosis in people living with HIV. The pathway prioritizes rapid molecular testing, urine LF-LAM in eligible patients, culture, imaging, and site-directed specimen collection according to clinical severity, immunological status, disease presentation, and specimen availability. In hospitalized or severely ill patients with advanced HIV disease, complementary tests should preferably be performed in parallel to reduce diagnostic delay. Bronchoscopy, bronchoalveolar lavage, EBUS-TBNA, biopsy, and histopathology remain important when non-invasive testing is inconclusive or morphological confirmation is required. Dashed borders indicate emerging or investigational technologies not yet incorporated into routine diagnostic recommendations. A negative result from a single test should not be interpreted as excluding TB when clinical suspicion remains high [5,7,8,9,17,18,24,27]. Abbreviations: ADA, adenosine deaminase; BAL, bronchoalveolar lavage; CAD, computer-aided detection; CRP, C-reactive protein; EBUS-TBNA, endobronchial ultrasound-guided transbronchial needle aspiration; HIV, human immunodeficiency virus; IFN-γ, interferon-gamma; LF-LAM, lateral-flow urine lipoarabinomannan assay; TB, tuberculosis.

Programmatically, a reasonable strategy for resource-limited settings would be to prioritize high-impact tests in the highest-risk groups. For example, in hospitalized patients with advanced HIV disease, LF-LAM should be available from admission; in patients with suspected pulmonary disease, Xpert Ultra should be the initial molecular test; in active case-finding contexts, CAD or radiography may function as screening; and in patients with suspected extrapulmonary disease, samples from the affected site should be processed with molecular tests and culture when possible. This approach allows resources to be allocated according to clinical risk and probability of benefit [18,24,27]. The proposed hierarchy is summarized as a rapid scenario-based clinical pathway in Figure 1, while Table 4 provides more detailed recommendations, complementary tests, and interpretative precautions for each clinical scenario.

## 11. Future Perspectives

The future of TB-HIV diagnosis will probably depend on multimodal algorithms that integrate clinical data, molecular tests, urinary antigens, blood biomarkers, digital imaging, and artificial intelligence. Instead of seeking a single universal test, the field appears to be moving toward combinations adapted to specific scenarios: hospitalization, outpatient care, advanced disease, suspected extrapulmonary TB, active case finding, or resistance detection [7,18].

FujiLAM II represents a promising development in non-sputum-based TB diagnosis because it was specifically redesigned to address the lot-to-lot variability observed with the original FujiLAM assay. Recent studies suggest improved manufacturing consistency and higher sensitivity than LF-LAM in some populations; however, performance estimates remain heterogeneous and do not establish uniform superiority over the original FujiLAM assay. Its programmatic incorporation will require additional prospective validation using fresh urine specimens, assessment across diverse clinical settings, commercial availability, cost-effectiveness analysis, and evaluation of its effect on treatment initiation and patient outcomes [10,30,31,32,33].

Molecular tests in non-respiratory samples are also a priority line of research. The use of Xpert Ultra or similar platforms in urine, blood, stool, oral swabs, or extrapulmonary samples could improve detection in people with advanced HIV disease or those unable to produce sputum. However, these approaches must be carefully evaluated to determine in which patients, samples, and scenarios they provide the greatest diagnostic value [18].

Host biomarkers and multiprotein signatures could be incorporated as triage tests, especially if they can be adapted to low-cost point-of-care platforms. Their role would not be to confirm TB, but rather to identify patients who require priority confirmatory evaluation. Future research should focus on prospective validation, capillary samples, populations with advanced HIV disease, coinfections, and settings with a high burden of comorbidities [11,12].

Artificial intelligence applied to chest radiography will also continue to evolve. Its greatest potential lies in large-scale screening programs, primary care, and regions with shortages of radiologists. However, its use in people with HIV requires specific validation, local threshold selection, and performance evaluation in paucibacillary or radiographically atypical TB. In addition, CAD systems must be integrated with confirmatory tests to avoid both false positives and clinically relevant false negatives [13,14].

Finally, the value of diagnostic innovation will depend on its ability to improve clinical decision-making and patient outcomes through timely diagnosis, prompt treatment initiation, resistance detection, and effective integration into health systems [7,18,24,27].

## 12. Conclusions

The diagnosis of TB in PLHIV remains challenging because immunosuppression alters clinical presentation, reduces bacillary burden, and increases extrapulmonary and disseminated disease. Consequently, conventional diagnostic methods remain important but are insufficient when used in isolation, particularly in patients with advanced immunosuppression [7,18].

Rapid molecular assays, particularly Xpert MTB/RIF and Xpert Ultra, have substantially improved microbiological confirmation and early detection of rifampicin resistance. However, their interpretation must always be integrated with clinical findings, imaging, culture, and non-sputum-based diagnostic approaches when appropriate [7,8].

Urine-based LAM tests occupy a central place in the diagnosis of advanced TB-HIV because they allow rapid evaluation of severely ill or hospitalized patients and those unable to produce sputum. Although LF-LAM has moderate sensitivity, its clinical usefulness is supported by evidence of reduced mortality in selected high-risk populations and by its incorporation into current WHO diagnostic recommendations. The original FujiLAM assay demonstrated greater sensitivity in early studies but was limited by clinically important lot-to-lot variability. FujiLAM II appears to improve manufacturing consistency and may increase diagnostic yield compared with LF-LAM in some settings, but further prospective and programmatic evaluation is required before widespread clinical adoption [5,10,24,27,28,29,30,31,32,33].

Emerging technologies, such as blood biomarkers, multiprotein signatures, transcriptomics, and artificial intelligence applied to chest radiography, should be considered complementary tools for triage and diagnostic prioritization. Their role is not to replace microbiological or molecular confirmation, but to help identify patients who require additional evaluation, especially in settings with high diagnostic demand or limited access to specialized tests. Their implementation requires population-specific validation before widespread clinical adoption [11,13].

In conclusion, the future of TB diagnosis in PLHIV will not depend on a single test, but on integrated, flexible algorithms adapted to the clinical scenario. In hospitalized patients or those with advanced HIV disease, tests should be applied in parallel and oriented toward reducing mortality; in outpatients, they should balance sensitivity, specificity, and rational use of resources; and when extrapulmonary or disseminated disease is suspected, diagnostic evaluation should extend beyond sputum. Ultimately, the greatest impact of future diagnostic innovation will not derive from any single technology, but from the rational integration of complementary diagnostic tools into patient-centered diagnostic pathways that enable earlier diagnosis, timely treatment initiation, resistance detection, reduced losses along the continuum of care, and ultimately fewer preventable deaths [7,18].

## Figures and Tables

**Table 1:** Key diagnostic challenges of tuberculosis in people living with HIV.

Diagnostic Challenge	Mechanism in People Living with HIV	Effect on TB Diagnosis	Practical Implication
Atypical or nonspecific clinical presentation	HIV-associated immunosuppression modifies the inflammatory response and may reduce the frequency of classic pulmonary symptoms.	TB may present with fever, weight loss, anemia, functional decline, sepsis-like syndrome, or overlapping manifestations of other opportunistic infections.	Clinical suspicion should remain high even when classic respiratory symptoms are absent or incomplete [2,7].
Limited performance of symptom-based screening	The World Health Organization four-symptom rule may perform differently according to antiretroviral therapy status and degree of immunosuppression.	Absence of cough, fever, weight loss, or night sweats does not reliably exclude active TB in PLHIV.	Symptom screening should be combined with microbiological, molecular, radiological, or non-sputum-based tests when clinical risk persists [15].
Paucibacillary disease	Lower bacillary load in respiratory secretions is more frequent as CD4+ T-cell counts decline.	Sputum smear microscopy sensitivity decreases, and microbiological confirmation may be delayed or missed.	A negative sputum smear result should not be used to rule out active TB in PLHIV, especially in advanced immunosuppression [7,16].
Difficulty obtaining adequate samples	Patients with advanced HIV disease may be unable to produce sputum or may have disease predominantly outside the lung.	Diagnosis may require less accessible samples, such as pleural fluid, cerebrospinal fluid, lymph node aspirates, biopsies, or blood.	Diagnostic algorithms should include alternative specimens and non-sputum-based tests when respiratory samples are unavailable or insufficient [3,17].
Extrapulmonary disease	HIV-related immunosuppression increases the likelihood of lymph node, pleural, central nervous system, abdominal, genitourinary, or other extrapulmonary involvement.	Sputum-based diagnostic pathways may fail to detect disease when the primary site is outside the respiratory tract.	Site-directed sampling, molecular testing of extrapulmonary specimens, imaging, culture, and clinical judgment are required [17,18].
Disseminated or severe TB	Advanced HIV disease may favor systemic spread of *M. tuberculosis* and severe disease with nonspecific systemic manifestations.	TB may mimic sepsis or other opportunistic infections, and initial sputum or radiographic tests may be negative.	Rapid, parallel diagnostic strategies are needed, including urine LAM, molecular tests, culture, imaging, and evaluation of non-respiratory samples [18].
Atypical or normal chest radiography	Advanced immunosuppression may reduce cavitation and produce diffuse, lower zone, miliary, lymph node, pleural, or subtle radiographic findings.	Chest radiography may lack specificity and may fail to rule out TB when findings are absent or atypical.	Radiography should be used as a screening or triage tool, not as a stand-alone confirmatory or exclusionary test [2,7].
Delays from conventional diagnostic pathways	Culture requires laboratory infrastructure and prolonged turnaround time; access to molecular tests may be uneven.	Treatment initiation may be delayed in patients with advanced HIV disease or severe disease.	Diagnostic pathways should prioritize rapid tests and parallel testing in high-risk patients to reduce delays and improve clinical decision-making [5,8].

This table summarizes the main clinical, microbiological, radiological, and operational barriers that complicate TB diagnosis in PLHIV. These challenges are not mutually exclusive and frequently coexist in patients with advanced immunosuppression. A negative result from a single diagnostic test should not be interpreted as excluding TB when clinical suspicion remains high. Abbreviations: HIV, human immunodeficiency virus; LAM, lipoarabinomannan; PLHIV, people living with HIV; TB, tuberculosis.

**Table 2:** Core, complementary, and near-implementation diagnostic tools for tuberculosis in people living with HIV.

Diagnostic Tool	Main Diagnostic Role	Key Strengths	Main limitations and Best-Use Scenario in People Living with HIV
Sputum smear microscopy	Initial or legacy microbiological test for pulmonary TB using sputum	Low cost, rapid, widely available, and useful in patients with high bacillary load	Low sensitivity in paucibacillary and smear-negative disease; does not detect drug resistance. It may be useful where molecular tests are unavailable or as support for infection control when bacillary load is high [7,16].
Mycobacterial culture	Microbiological confirmation and drug susceptibility testing using respiratory or extrapulmonary specimens	Higher sensitivity than smear microscopy; detects viable organisms; enables phenotypic drug susceptibility testing	Slow turnaround time and dependence on laboratory infrastructure. Best used for complex cases, suspected drug resistance, relapse, treatment failure, or extrapulmonary TB when samples are available [5,7].
Chest radiography	Screening, triage, and initial evaluation of pulmonary disease	Rapid, noninvasive, and useful when microbiological or molecular testing is delayed or unavailable	Limited specificity: findings may be atypical or absent in advanced immunosuppression. It should be used to prioritize confirmatory testing, not to confirm or rule out TB in isolation [7,13].
Xpert MTB/RIF	Rapid molecular detection of *M. tuberculosis* and rifampicin resistance	Automated, rapid, and more sensitive than smear microscopy; detects rifampicin resistance	May miss paucibacillary or smear-negative disease and depends on adequate sample availability. Useful as an initial molecular test where Xpert Ultra is unavailable [7,8].
Xpert MTB/RIF Ultra	High-sensitivity rapid molecular test for TB and rifampicin resistance	Greater sensitivity than Xpert MTB/RIF, especially in smear-negative TB and PLHIV; rapid rifampicin resistance detection	Trace-positive results require clinical interpretation, and specificity may be lower in patients with previous TB. Preferred initial molecular test in high-risk, smear-negative, or paucibacillary pulmonary TB [8,21].
LF-LAM/AlereLAM	Adjunctive urine-based rapid test for advanced TB-HIV	Noninvasive sample, bedside use, rapid result, and evidence of mortality benefit in high-risk hospitalized patients	Moderate sensitivity and no drug resistance information. Most useful in hospitalized patients with HIV, advanced immunosuppression, severe illness, low CD4+ counts, inability to produce sputum, or suspected disseminated TB [9,24,27].
FujiLAM/FujiLAM II	Emerging next-generation urine-based LAM assays	The original FujiLAM showed greater sensitivity than LF-LAM in early studies; FujiLAM II was redesigned to improve manufacturing consistency and has outperformed LF-LAM in some recent evaluations	The original assay showed clinically important lot-to-lot variability. FujiLAM II has more consistent inter-lot performance, but sensitivity remains variable across populations and reference standards, and it is not yet included in routine WHO diagnostic recommendations. Further prospective validation, cost-effectiveness assessment, quality assurance, and programmatic evaluation are required [10,28,29,30,31,32,33].

This table summarizes core, complementary, and near-implementation diagnostic tools for TB in PLHIV. Rapid molecular testing and urine LF-LAM in eligible PLHIV constitute priority diagnostic tools; culture and drug susceptibility testing provide confirmation and resistance characterization; smear microscopy, radiography, and invasive procedures have context-dependent supportive roles; and FujiLAM II remains an emerging assay requiring further programmatic validation. Test selection should consider clinical severity, degree of immunosuppression, specimen availability, pretest probability, and local diagnostic capacity. Abbreviations: HIV, human immunodeficiency virus; LF-LAM, lateral flow lipoarabinomannan assay; PLHIV, people living with HIV; TB, tuberculosis.

**Table 3:** Emerging triage and screening tools for tuberculosis in people living with HIV.

Tool or Approach	Sample or Data Source	Intended Role	Potential Advantages	Main Limitations in People Living with HIV	Practical Interpretation
C-reactive protein	Blood, including potential point-of-care or capillary blood platforms	Triage or initial rule-out tool	Simple, relatively accessible, high sensitivity in PLHIV, and potentially useful to prioritize confirmatory testing	Low specificity; may be elevated due to bacterial infections, fungal infections, malignancies, inflammation, or other HIV-associated conditions	Useful to identify patients who require Xpert Ultra, culture, urine LF-LAM, or radiological evaluation, but not sufficient to confirm TB [11,37,38,39].
IP-10	Blood or plasma	Candidate host-response biomarker for triage	Reflects interferon-mediated immune activation and may complement other inflammatory markers	Specificity may be reduced by chronic immune activation, opportunistic infections, or systemic inflammation in advanced HIV disease	Promising but investigational; requires validation in populations stratified by CD4+ count, antiretroviral therapy status, and coinfections [11,37].
NCAM-1 and SAA	Blood or plasma	Candidate protein biomarkers or components of multiprotein panels	May improve performance when combined with other host-response markers	Evidence remains heterogeneous; cut-off points, platforms, and populations vary across studies	Should be considered research tools until prospective validation and operational standardization are available [11].
Multiprotein signatures	Blood-based biomarker panels	Triage, diagnostic prioritization, or risk stratification	Integrate several inflammatory or immune-response pathways and may improve performance compared with single biomarkers	Require platform development, validation, cost evaluation, and assessment in HIV-associated comorbidities	Potentially useful for future point-of-care triage algorithms, but not ready to replace confirmatory microbiological or molecular testing [11].
Transcriptomic signatures	Blood RNA expression profiles	Triage, diagnosis, or prediction of progression to active TB	Capture host-response patterns and may identify TB before conventional clinical confirmation in selected contexts	Technologically complex; may be affected by HIV-related immune dysregulation and other infections; limited real-world validation in advanced HIV disease	Promising for future diagnostic platforms, but current use remains mainly investigational in TB-HIV [12].
Chest radiography with CAD	Digital chest radiograph	Screening or triage tool	Rapid, scalable, useful where expert radiologists are scarce, and potentially applicable in active case-finding programs	Sensitivity may decrease in PLHIV and in smear-negative or radiographically atypical disease; specificity is moderate	Can prioritize confirmatory testing, but a negative CAD result should not rule out TB when clinical suspicion remains high [13,14,41].
CAD systems such as CAD4TB, Lunit, and qXR	Digital chest radiograph analyzed by artificial intelligence algorithms	Automated radiographic screening	Provide standardized TB probability scores and may support high-volume screening programs	Performance varies by software, threshold, population, HIV status, and smear status; thresholds require local validation	Should be implemented with predefined thresholds, local validation, and clear pathways for Xpert Ultra, culture, or clinical evaluation [13,14].

Within the proposed hierarchy, CRP and computer-aided chest radiography may be used as triage tools when their performance and thresholds have been locally validated, but they should not be interpreted as stand-alone confirmatory tests. IP-10, NCAM-1, SAA, multiprotein panels, and transcriptomic signatures remain investigational and should not replace molecular or microbiological confirmation. In PLHIV, especially those with advanced immunosuppression, inflammatory biomarkers and radiographic algorithms may be affected by opportunistic infections, atypical radiological patterns, and paucibacillary disease. Abbreviations: CAD, computer-aided detection; CRP, C-reactive protein; HIV, human immunodeficiency virus; IP-10, interferon gamma-induced protein 10; LAM, lipoarabinomannan; NCAM-1, neural cell adhesion molecule 1; PLHIV, people living with HIV; SAA, serum amyloid A; TB, tuberculosis.

**Table 4:** Proposed integrated diagnostic algorithm for tuberculosis in people living with HIV.

Clinical Scenario	Priority Initial Evaluation	Context-Dependent Complementary Tests	Interpretation and Precautions
Hospitalized patient with advanced HIV disease or signs of severity	Immediate clinical assessment, chest radiography, urine LF-LAM, and Xpert Ultra in sputum if the patient can expectorate	Mycobacterial culture, extrapulmonary samples according to suspected site, and drug susceptibility testing	Prioritize parallel testing. A single negative result does not rule out TB if clinical suspicion is high. Therapeutic decisions should not be delayed in severe disease [18,24,27].
Outpatient with HIV and suspected pulmonary TB	Clinical screening, CRP if available, chest radiography or CAD, and Xpert Ultra as the initial molecular test	Culture if Xpert Ultra is negative but suspicion persists; urine LF-LAM in advanced HIV disease, low CD4+ count, or systemic symptoms	CRP and CAD function as triage tools, not confirmatory tests. Xpert Ultra remains a priority when an adequate respiratory sample is available [8,11,13].
Suspected extrapulmonary TB	Clinical evaluation directed to the affected site and collection of a sample from the suspected site whenever possible	Xpert Ultra, culture, histopathology, targeted imaging, and urine LF-LAM if advanced HIV disease or hospitalization is present	A negative sputum result does not exclude extrapulmonary disease. Prioritize samples from the affected site and interpret results in the clinical context [7,17,42].
Suspected disseminated TB or sepsis-like syndrome in advanced HIV disease	Severity assessment, urine LF-LAM, chest radiography, and Xpert Ultra if a sample is available	Mycobacterial blood culture where available, culture of other samples, and molecular tests in non-respiratory samples	Consider TB even without positive sputum. Disseminated disease may require early treatment based on clinical risk and severity [18].
Suspected tuberculous meningitis	Urgent neurological evaluation, lumbar puncture if not contraindicated, and cerebrospinal fluid analysis	Xpert Ultra and culture in cerebrospinal fluid, brain imaging, and evaluation of differential diagnoses	A negative molecular test in cerebrospinal fluid does not exclude tuberculous meningitis. If clinical suspicion is high, treatment should not be delayed [17].
Suspected drug resistance	Xpert MTB/RIF or Xpert Ultra for initial detection of rifampicin resistance	Xpert MTB/XDR, line probe assays, culture with phenotypic drug susceptibility testing, or targeted sequencing when available	Rifampicin resistance is an important initial marker, but it does not assess the full resistance profile. Expand testing if there is previous treatment, treatment failure, contact with drug-resistant TB, or high epidemiological suspicion [5,8,18,52].
Active case-finding or community screening programs	Symptom screening, chest radiography or CAD, and CRP where available	Xpert Ultra for confirmation; culture according to availability; urine LF-LAM if advanced HIV disease or systemic symptoms are present	CAD and CRP help prioritize testing but require local validation and subsequent microbiological or molecular confirmation [11,13,14].

This table proposes a practical, hierarchical, and scenario-based approach to TB diagnosis in PLHIV. Diagnostic priority reflects current guideline support, strength of clinical evidence, capacity to produce timely and actionable results, specimen availability, and applicability to the specific clinical presentation. It is not intended as a rigid sequence of tests, because context-dependent complementary methods may become immediate priorities in extrapulmonary, disseminated, neurological, or severely ill presentations. In advanced HIV disease, especially in hospitalized or severely ill patients, parallel testing is preferred over sequential testing to reduce diagnostic delays. Abbreviations: CAD, computer-aided detection; CRP, C-reactive protein; HIV, human immunodeficiency virus; LF-LAM, lateral flow lipoarabinomannan assay; LAM, lipoarabinomannan; PLHIV, people living with HIV; TB, tuberculosis.

## Data Availability

No new data were created or analyzed in this study. Data sharing is not applicable to this article.

## Abbreviations

The following abbreviations are used in this manuscript:

ADA	Adenosine deaminase
ART	Antiretroviral therapy
BAL	Bronchoalveolar lavage
CAD	Computer-aided detection
CAD4TB	Computer-aided detection for tuberculosis
CD4	Cluster of differentiation 4
COVID-19	Coronavirus disease 2019
CRP	C-reactive protein
CSF	Cerebrospinal fluid
DST	Drug susceptibility testing
EBUS-TBNA	Endobronchial ultrasound-guided transbronchial needle aspiration
FujiLAM	Fujifilm SILVAMP TB-LAM
FujiLAM II	Updated Fujifilm SILVAMP TB LAM II assay
HIV	Human immunodeficiency virus
IFN-γ	Interferon-gamma
IP-10	Interferon gamma-induced protein 10
LAM	Lipoarabinomannan
LF-LAM	Lateral-flow urine lipoarabinomannan assay
MGIT	Mycobacterial growth indicator tube
NCAM-1	Neural cell adhesion molecule 1
PLHIV	People living with HIV
PTB	Pulmonary tuberculosis
qXR	qXR computer-aided chest radiography software
SAA	Serum amyloid A
TB	Tuberculosis
WHO	World Health Organization
Xpert MTB/RIF	Xpert MTB/RIF assay
Xpert MTB/RIF Ultra	Xpert *Mycobacterium tuberculosis*/rifampicin Ultra assay
Xpert MTB/XDR	Xpert MTB/XDR assay
